# Predictive Value of Cardiac Magnetic Resonance for Left Ventricular Remodeling of Patients with Acute Anterior Myocardial Infarction

**DOI:** 10.3390/diagnostics12112780

**Published:** 2022-11-14

**Authors:** Wenkun Ma, Xinni Li, Chengjie Gao, Yajie Gao, Yuting Liu, Sang Kang, Jingwei Pan

**Affiliations:** 1 Department of Cardiovasology, Shanghai Sixth People’s Hospital Affiliated to Shanghai Jiao Tong University School of Medicine, Shanghai 200233, China; 2 Department of Geriatrics, Shanghai Sixth People’s Hospital Affiliated to Shanghai Jiao Tong University School of Medicine, Shanghai 200233, China

**Keywords:** acute myocardial infarction, cardiac magnetic resonance, left ventricular remodeling, strain

## Abstract

Background: Heart failure is a serious complication resulting from left ventricular remodeling (LVR), especially in patients experiencing acute anterior myocardial infarction (AAMI). It is crucial to explore the predictive parameters for LVR following primary percutaneous coronary intervention (PPCI) in patients with AAMI. Methods: A total of 128 AAMI patients who were reperfused successfully by PPCI were enrolled sequentially from June 2018 to December 2019. Cardiovascular magnetic resonance (CMR) was performed at the early stage (<7 days) and after the 6-month follow-up. The patients were divided into LVR and non-LVR groups according to the increase of left ventricular end diastolic volume (LVEDV) measured by the second cardiac magnetic resonance examination ≥20% from baseline. (3) Results: The left ventricular ejection fraction (LVEF), the global longitudinal strain (GLS), the peak circumferential strain in infarcted segments, and the infarct size (IS) remained significantly different in the multivariate logistic regression analysis (all *p* < 0.05). The area under the receiver operating characteristic curve of Model 1, wherein the GLS was added to the LVEF, was 0.832 (95% CI 0.758–0.907, *p* < 0.001). The C-statistics for Model 2, which included the infarct-related regional parameters (IS and the peak circumferential strain in infarcted segments)was 0.917 (95% CI 0.870–0.965, *p* < 0.001). Model 2 was statistically superior to Model 1 in predicting LVR (IDI: 0.190, *p* = 0.002). (4) Conclusions: Both the global and regional CMR parameters were valuable in predicting LVR in patients with AAMI following the PPCI. The local parameters of the infarct zones were superior to those of the global ones.

## 1. Introduction

Acute myocardial infarction (AMI) can lead to irreversible myocardial necrosis. Al-though the mortality at the acute stage has been notably reduced by primary percutaneous coronary intervention (PPCI), the instances of long-term adverse events, especially chronic heart dysfunction, remain high [1]. Necrotic myocardium impairs cardiac contractile function with a decreased ejection fraction and compensatory cardiac dilatation, which attributes to refractory heart failure, especially acute anterior myocardial infarction (AAMI). AAMI is caused by the acute block of the left anterior descending coronary artery (LAD), which perfuses the anterior septum and anterior wall of the left ventricle (LV). Under the similar myocardial salvage, the patients with AAMI have a larger infarcted size than the non-AAMI patients do [2], which more likely leads to adverse left ventricular remodeling (LVR). Predicting LVR after AAMI is crucial because LVR is closely related to an adverse prognosis. 

The previous risk stratification of AMI, such as Global Registry of Acute Coronary Events (GRACE) and Thrombolysis in Myocardial Infarction (TIMI) scores [3,4], which are established before a routine PPCI, provide limited indicative power in patients with AMI that is treated with PPCI. The parameters that are included in the previous risk stratification cannot precisely quantify the damage of the LV. Cardiovascular Magnetic Resonance (CMR) has been conclusively demonstrated as an accurate and effective method for identifying patients who are suffering from AMI with poor clinical outcomes [5,6]. CMR can provide a comprehensive assessment of the functional and structural damage that is caused by AMI [7]. Standard parameters such as the left ventricular ejection fraction (LVEF) and the infarct size based on gadolinium-enhanced sequences in the early stages of AMI are valuable prognostic factors [8]. While the myocardial strain is another informative parameter that can reveal cardiac deformation both globally and within the MI zone [9,10]. Early studies have explored the CMR multiparameter risk score as a prognostic indicator [11,12]. Moreover, it is still unclear whether adverse LVR is associated with the loss of viable myocardium in the MI zone or excessive compensation from the non-MI region. Which factors are more effective in predicting the outcomes in patients with AAMI: the global parameters or the regional ones? The global parameters consider the integrated function of the left ventricle, while the regional parameters are focused on the influence of the infarct zones [13]. Hence, we conducted this study to explore the delineated question.

## 2. Patients and Methods

### 2.1. Study Population and Data Collection

This prospective study was performed between June 2018 and December 2019 at the cardiology center of the Shanghai Sixth People’s Hospital. The enrolled patients were diagnosed with AAMI and treated with PPCI. The study inclusion criteria were as follows: (1) AMI which was diagnosed according to the AMI diagnostic criteria [14] (i.e., clinical symptoms of myocardial ischemia lasting <12 h, ST segment elevation >0.2 mV in ≥2 adjacent precordial leads, and/or an acute myocardial biomarker serum troponin level >99% of the upper limit of the normal value); (2) PPCI which was performed within 12 h after the onset of chest pain (the PPCI window was extended to 36 h if the patients had endured hemodynamic instability, such as in cardiogenic shock); (3) the culprit coronary artery that caused myocardial infarction was the LAD. Study exclusion criteria were as follows: (1) non-ST segment elevation myocardial infarction or previous myocardial infarction; (2) active myocarditis or cardiomyopathy (i.e., dilated cardiomyopathy, hypertrophic cardiomyopathy, or restrictive cardiomyopathy); (3) valvular disease of moderate or greater severity; (4) cardiac pacemaker implantation; (5) malignant arrhythmia; (6) chronic obstructive pulmonary disease, severe anemia, malnutrition, severe kidney dysfunction, and/or an estimated glomerular filtration rate ≤30 mL/min/1.73 m^2^; (7) claustrophobia or magnetic resonance imaging contraindications (such as an allergy to the contrast agent). 

The patients underwent CMR examinations during both the early phase following AAMI (i.e., within 7 days of the onset) and after the six-month follow-up visit. Demographic data and medical history were collected from the patients in the study. The collected data included information on laboratory examinations, coronary vascular disease risk factors, concomitant diseases, and medicines. Ethical approval for this study was obtained from Shanghai Jiao Tong University which is affiliated to the Shanghai Sixth People’s Hospital (2017-KY-003 (K)). All of the patients provided written informed consent. 

### 2.2. Definition of LVR

The definition of LVR was an increase in left ventricular end-diastolic volume (LVEDV) at ≥20% over approximately six months of follow-ups (ΔLVEDV% ≥ 20%) [13].

### 2.3. CMR Technique

The ECG-gated CMR examinations were performed using a 3-T system (Philips Healthcare, Best, The Netherlands) within seven days of the onset and at the six-month follow-up visit. The CMR protocol included a localizer, T2-weighted, cine, first-pass perfusion, and delay-enhancement sequences. Each long-axis slice was subjected to balanced, steady-state free precision (bSSFP) cines (with a 60° separation around the long axis of the LV (two, three, and four chambers)). A total of 10–12 continuous slices of short-axis cines were obtained after the injection of the contrast agent (gadolinium, 0.2 mmol/kg), covering the entire LV region from the ring of the mitral valve to the apex (parallel slices 8 mm wide without gap; TE = 1.6 ms, TR = 3.2 ms, flip angle 45, voxel size 2.0 × 1.6 × 8 mm^3^, field of view 350 × 350 mm; 30 phases in each cardiac cycle). The gadolinium enhancement sequences were acquired 10 min after the contrast injection (Magnevist, Bayer Healthcare, Berlin, Germany).

### 2.4. CMR Analysis 

**Figure 1:**
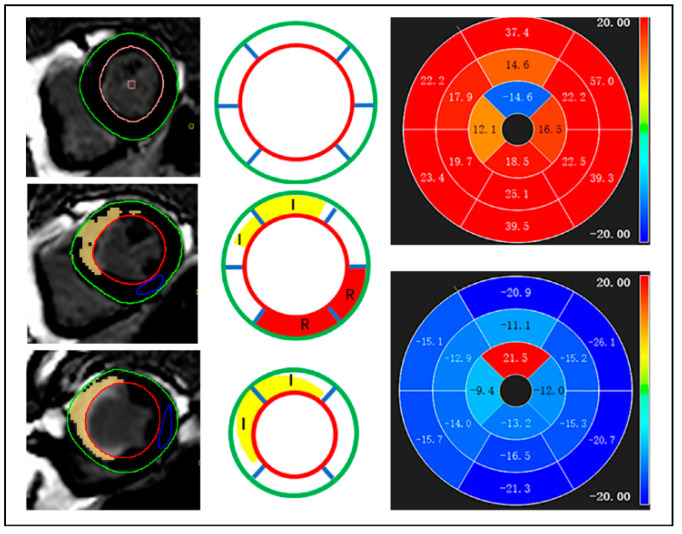
According to late gadolinium enhancement, the infarcted segments and remote non-infarcted segments were defined. Red and green were used to outline the endocardial and epicardial contours of the left ventricle, respectively. Myocardium was divided into infarcted (I, yellow) and remote non-infartced segments (R, red) based on the American Heart Association 16-segment mode after the healthy myocardium was delineated by a blue circle. Remote non-infarcted segments were defined as unenhanced zone with one unenhanced border segments between them and infarcted segments.

The LVEF, LV mass (LVM), LV end-diastolic volume (LVEDV), and LV end-systolic volume (LVESV) were analyzed using standardized protocols. The LVM was derived by subtracting the papillary muscles at the end diastole [15]. CVI42 (Circle Cardiovascular Imaging, Calgary, AB, Canada) was used for CMR feature tracking analysis of the bSSFP cine images. At the end diastole, the borders of the LV endocardium and epicardium were automatically tracked and manually corrected in the short-axis series with three long-axis slices. The long-axis cines were used to extract the global longitudinal strain (GLS) of the LV, whereas the short-axis cines were used to calculate the global circumferential strain (GCS) and global radial strain (GRS) [16,17]. Using the 16-segment American Heart Association model, the segments containing positive late gadolinium enhancement (LGE) were defined as infarct segments. The remote non-infarcted segments were identified as unenhanced segments which were separated from the infarct segments by a single unenhanced border segment. The regional peak strains were defined as the average of each segmental peak value [18] (Figure 1). The quantification of the infarct size (IS) was performed using LGE images which were obtained from the short axis. The IS was delineated using a semiautomated computer-aided threshold detection protocol (>5 standard deviations [SDs] of remote myocardium) and was calculated by dividing the sum of infarct size from all of the sections by the mass of LV areas (including those without infarct scar) and multiplying by 100.

### 2.5. Angiographic Assessments 

The angiographic analysis was performed following standard protocols, including those that have been delineated for TIMI [19]. All of the images were reviewed by a qualified interventional cardiologist (Prof. PAN).

### 2.6. Reproducibility Analysis

The data from 10 LVR patients and 10 non-LVR subjects were sampled to evaluate the inter- and intra-observer variability. The CMR analyses were performed independently by two cardiologists (Dr. MA and Dr. GAO) who were blinded to each other’s recordings. The inter-observer variability was tested using the data from different acquisitions. The observers reanalyzed their own recordings to check for intra-observer variability; these evaluations were conducted at 2 weeks apart.

### 2.7. Statistical Analysis

For the continuous variables, the means ± SD or medians and interquartile ranges (i.e., 25th to 75th percentiles) are reported, and the variable distributions were evaluated using the Kolmogorov–Smirnov test. Wilcoxon rank-sum tests was used for inter-group comparisons of the non-normally distributed variables, whereas the *t*-tests were used for inter-group comparisons of the normally distributed continuous variables. Fisher’s exact tests or chi-square tests were used to compare the categorical variables. The results are displayed as numbers and percentages. Moreover, univariate and multivariate logistic regression analyses were conducted to identify the prognostic predictors of LVR. A receiver operating characteristic (ROC) curve analysis was used to identify the parameters that were best suited for checking the LVR model. The optimal cut-off values were determined according to the maximum Youden index. The Delong test was used to compare the areas under the ROC curves (AUCs). We also calculated integrated discrimination improvement (IDI) by comparing global and regional parameters in order to find the strongest predictors of LVR following AAMI. The model calibration was assessed using the Hosmer–Lemeshow goodness-of-fit test, and the inter- and intra-observer reproducibility were determined using intraclass correlation coefficients. Standard statistical software (i.e., SPSS Statistics Version 26.0 (IBM Corp. Armonk, NY, USA), R version 4.2.0 (R Foundation for Statistical Computing, Vienna, Austria), and GraphPad Prism 9 (GraphPad Software, San Diego, CA, USA)) were used to perform all of the calculations. A two-sided *p*-value of <0.05 was considered to be the threshold for statistical significance.

## 3. Results

### 3.1. Patient Characteristics

Of the 128 included study participants, five of them were excluded due to them having a poor CMR image quality, and seven patients did not complete the follow-up. Consequently, 116 patients with AAMI were included in the final analysis; these patients were divided into an LVR group (*n* = 39) and a non-LVR group (*n* = 77). The average age was 58.22 ± 12.30 years, and 86.20% of the included patients were male. The patients in the LVR group had a higher serum level of peak hypersensitive cardiac troponin I (hs-cTnI). There were no statistically significant differences in the sex, age, heart rate, body mass index, pain to balloon time, coronary heart disease risk factors, Killip classification at the point of admission, peak pro-brain natriuretic peptide (pro-BNP) levels, TIMI flow pre-PPCI, TIMI flow after PPCI, and medication use between the study groups (all *p* > 0.05). The demographic and clinical characteristics of the included patients with AAMI are shown in Table 1. 

### 3.2. CMR Parameter Analysis

In this study, the initial CMR examinations were performed at 3 days (interquartile range, IQR: 2–5 days) after the AAMI onset, and the follow-up CMR tests were conducted at an average of 10 months (IQR: 8–12 months) after the AAMI onset. Compared with the non-LVR group, the patients who developed LVR presented with a lower LVEF and a more extensive IS. The GLS and the GCS of LV were statistically significantly decreased in the LVR group, while there was no difference in LVEDV, LVESV, and GRS. Using a segment strain analysis, the peak radial strain in infarcted segments (${\mathrm{RS}}_{\mathrm{infarct}}$) and the peak circumferential strain in infarcted segments (${\mathrm{CS}}_{\mathrm{infarct}}$) were statistically significantly lower in the LVR group (*p* < 0.001). There were no differences in the peak circumferential strain in remote non-infarcted segments (${\mathrm{CS}}_{\mathrm{remote}}$) or the peak radial strain in remote non-infarcted segments (${\mathrm{RS}}_{\mathrm{remote}}$) between the study groups. The CMR characteristics are listed in Table 2.

### 3.3. Cardiovascular Outcomes

The results of univariate and multivariate logistic regression analyses (i.e., in regard to the predictors of LVR) are presented in Table 3. In the univariate analysis, the predictors associated with LVR occurrence were as follows: peak hs-cTnI, LVEF, GLS, GCS, peak ${\mathrm{CS}}_{\mathrm{infarct}}$, peak ${\mathrm{RS}}_{\mathrm{infarct}}$, and IS (all *p* < 0.001). The peak ${\mathrm{CS}}_{\mathrm{infarct}}$ was highly correlated with the GCS and peak ${\mathrm{RS}}_{\mathrm{infarct}}$, (r = 0.72, *p* = 0.006; r = 0.805, *p* < 0.001). Therefore, the multivariable models included the following parameters: LVEF, GLS, peak ${\mathrm{CS}}_{\mathrm{infarct}}$, and IS. After testing, these four parameters (LVEF, GLS, ${\mathrm{CS}}_{\mathrm{infarct}}$, and IS) independently predicted the occurrence of LVR in patients with AAMI (*p* < 0.05).

### 3.4. ROC Curve Analysis of the Risk Score Model

**Figure 2:**
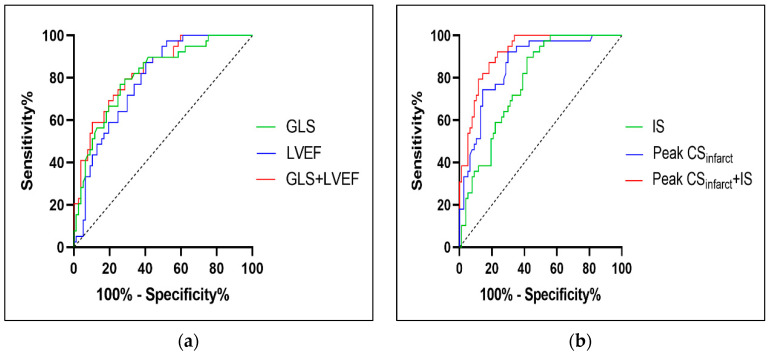
The area under ROC curves of the global parameters and regional parameters as a marker to predict LVR. (**a**) The AUCs of the LVEF, GLS, GLS + LVEF for predicting the occurrence of were 0.789 (95% CI 0.708–0.870), 0.812 (95% CI 0.731–0.893), and 0.832 (95% CI 0.758–0.907), respectively, all *p* < 0.001. (**b**) The AUCs of ${\mathrm{CS}}_{\mathrm{infarct}}$, IS, IS+${\mathrm{CS}}_{\mathrm{infarct}}$ for predicting the occurrence of were 0.861 (95% CI 0.793–0.930), 0.778 (95% CI 0.696–0.860), and 0.917 (95% CI 0.870–0.965), respectively, all *p* < 0.001. ROC, receiver operating characteristic; LVR, left ventricular remodeling; AUCs, the area under ROC curves; LVEF, left ventricular ejection fraction; GLS, global longitudinal strain; ${\mathrm{CS}}_{\mathrm{infarct}}$, circumferential strain in infarcted segments; IS, infarct size.

To predict LVR in the early stages of AAMI, four independent influencing factors of CMR were allocated between the models in order to construct two predictive models. Model 1, which included LVEF and GLS, evaluated the global change in functionality following AAMI, while Model 2, consisting of peak ${\mathrm{CS}}_{\mathrm{infarct}}$ and IS, emphasized the impairment of the infarct segments. In Model 1, the GLS predicted LVR with an AUC of 0.812 (95% CI 0.731–0.893). The addition of the GLS to the LVEF resulted in a better prognostic value and an improved risk evaluation, with an increase in the AUC from the sole LVEF evaluation (AUC, 0.789, 95% CI 0.708–0.870) to the LVEF + GLS (AUC, 0.832, 95% CI 0.758–0.907) (Figure 2a). In Model 2, the AUC for the peak ${\mathrm{CS}}_{\mathrm{infarct}}$ and IS in regard to LVR in the patients with AAMI were 0.861 (95% CI 0.793–0.930) and 0.778 (95% CI 0.696–0.860), respectively. After combining the two parameters (${\mathrm{CS}}_{\mathrm{infarct}}$+IS), the AUC increased to 0.917 (95% CI 0.870–0.965) (Figure 2b). Each model passed the Hosmer–Lemeshow goodness-of-fit test (all *p* > 0.05).

### 3.5. Incremental Effects of Global and Regional Parameters

**Figure 3:**
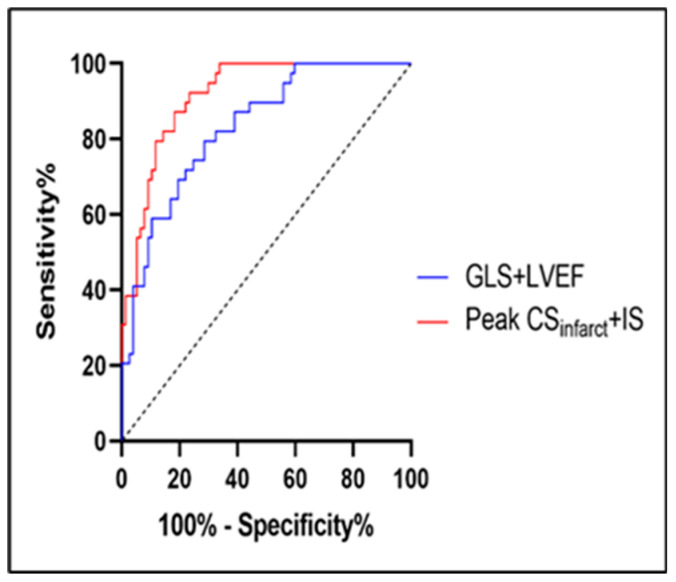
Discriminative prognostic power of GLS + LVEF and peak ${\mathrm{CS}}_{\mathrm{infarct}}$+IS. Area under ROC curves of GLS + LVEF in comparison to peak ${\mathrm{CS}}_{\mathrm{infarct}}$+IS for the prediction of LVR. Peak ${\mathrm{CS}}_{\mathrm{infarct}}$+IS revealed a significantly higher AUC than GLS + LVEF did (0.917, 95% CI: 0.870–0.965 *p* < 0.001 vs. 0.832, 95% CI 0.758–0.907, *p* < 0.001. AUC difference: 0.085, *p* < 0.05). GLS, global longitudinal strain; LVEF, left ventricular ejection fraction; ${\mathrm{CS}}_{\mathrm{infarct}}$, circumferential strain in infarcted segments; IS, infarct size; ROC, receiver operating characteristic; LVR, left ventricular remodeling; AUC, the area under ROC curve.

Figure 3 suggests that when it was compared with Model 1 (LVEF + GLS), Model 2 (${\mathrm{CS}}_{\mathrm{infarct}}$+IS) showed a better prognostic value for LVR based on the Delong test (*p* < 0.05). Moreover, Model 2 demonstrated statistically improved discrimination and reclassification, with an IDI of 0.190 (*p* = 0.002).

### 3.6. Reproducibility 

We found good intra- and inter-observer variabilities for the evaluated CMR measurements. Supplement Table S1 and Supplement Figure S1 show the relevant detailed Bland–Altman graphics and the correlation coefficient data.

## 4. Discussion

This study focused on the prediction of adverse LVR with CMR parameters. LVR is a major cause of heart failure following AMI. Strain imaging can detect early or subtle changes in LV function and can effectively predict the AMI prognosis. This study revealed that the analysis of both the regional and global parameters in regard to myocardial strain is valuable for predicting LVR in patients with AAMI who are undergoing PPCI. By investigating two predictive models further, which were composed of the CMR parameters, we found that Model 2, which combined peak ${\mathrm{CS}}_{\mathrm{infarct}}$ and IS, manifested more predictive power than Model 1 did, which included a combination of global parameters, including LVEF and GLS. The parameters of the MI zone were more efficient and specific for LVR prediction following AAMI in the present study. 

To evaluate LVR following AMI, we investigated AAMI that was caused by LAD occlusion in order to avoid confusing different myocardial injuries that are perfused by different coronary arteries. LVR resulting from AAMI impairs the heart function more severely than the remodeling of non-AAMI, and even the level of myocardial necrosis biomarkers serum troponin I are the same [2]. All of the patients in this study underwent PPCI treatment. Revascularization over time can save more myocardium which is helpful to reserve the heart contraction, on the other hand, oxygen free radicals releasing from successful reperfusion may aggravate myocardial edema or injury. Many factors, such as the severity of the local ischemia, local deformation, local hormone, stress, and autophagy, affect the myocyte salvage and collagen synthesis. Following AMI, the contraction becomes imbalanced and unsynchronized [20]. LVEF is reduced because the loss of vital myocyte is replaced with the abnormal distribution of collagen, meanwhile the reserved myocardium compensates the need for circulation by accelerating the segmental motion in non-infarcted segments, which also changes the mechanical properties of remote non-infarcted myocardium. This progressively contributes to negative remodeling [20,21]. LVR following AMI represents a compensatory balance between the infarcted and non-infarcted regions, and the global parameters may efficiently integrate the changes in the infarcted and non-infarcted regions. LVEF is a well-known factor contributing to systolic function. Additionally, it has been associated with a poor prognosis in patients with AMI [22]. However, LVEF had a limited value in predicting the patient mortality when it was above 45% [23]. Several studies have verified that the GLS is an important indicator of the prognosis of AMI [10,24]. More importantly, this study revealed that the addition of GLS to LVEF in a prediction model improved its predictive power in regard to LVR following AAMI. It seems that GLS detects more potential damage as well as subclinical damage in regard to LV function when it is compared with LVEF, which has been reported by Altiok and Ben Driss [25,26]. 

This study assessed both the global and segmental strain using the feature tracking module of CVI42, and we have identified GLS as a more accurate indicator of LVR than LVEF is. However, the factors describing the circumferential motion of the infarct zones seemed to predict LVR more effectively than GLS did in the present study. GCS is well recognized as a determinant of AMI prognosis based on the findings of two other studies. More specifically, Mangion’s team found that the circumferential strain was an independent prognostic factor in patients with ST-segment elevation myocardial infarction [27], and Buss et al. revealed that GCS could predict LVEF alterations at six months following AMI [28]. There are three layers of myocardium in the left ventricle: the oblique inner and outer layers and the circular middle layer. The circumferential myofibers are less predisposed to ischemia and a subsequent infarction owing to its anatomic location. The inner layer of the myocardium moves longitudinally during systole, whereas the middle and outer layers move circumferentially. Therefore, the middle and outer layers have a greater influence on the LV circumferential motion, which may help to maintain the shape and size of the LV, and it may serve as a functional biomarker of myocardial salvage and the propensity to reserve the LV pump function in the long term [27]. Circumferential motion can cause LVR due to the degree of damage. We found that peak ${\mathrm{CS}}_{\mathrm{infarct}}$ and IS could predict LVR better than LVEF and GLS could (IDI, 0.190 (0.0712–0.3089) *p* = 0.002). The evidence demonstrated that the myocardial biomechanical changes that follow MI were initially limited to the MI region, but they gradually extended to the neighboring border zones and the remote myocardium over the 28 days post-MI [29]. This might account for why the regional parameters are more sensitive than the global one in predicting the LVR in patients with AAMI. In addition, we found that the peak ${\mathrm{CS}}_{\mathrm{infarct}}$+ LVEF showed a similar predictive power as the peak ${\mathrm{CS}}_{\mathrm{infarct}}$+IS did (Supplement Figure S2); the former combination will be available for patients with impaired renal function who undergo an abbreviated CMR protocol without contrast.

It is vital to explore the predictors of LVR in regard to the clinical adverse outcomes in patients with AMI. This information would be extremely helpful for patients in the high-risk group, who could receive further novel therapies in addition to routine post-AMI medications, as well as more aggressive up-titration of prognostic medicines (e.g., beta blockers, ACEI, and mineralocorticoid receptor antagonists) and more in-depth follow-ups in order to identify an adverse LVR. For example, this information could aid the prescription of primary prevention implantable cardioverter-defibrillators which aim to reduce the major clinical events that are associated with AMI.

## 5. Limitations

We acknowledge several limitations of the present research. Firstly, this was a single-center study, and therefore, the generalizability of our findings is unclear. Moreover, only 128 patients were diagnosed with AAMI and treated with PPCI. Hence, the enrolled sample size was small, which might have affected the accuracy of the cutoff values that were obtained in the multivariate analysis. The sample size should be expanded to obtain more reliable clinical conclusions, and the follow-up time should be extended. New parameter such as the sphericity index of LV will be explored in future studies.

## 6. Conclusions

The current study demonstrated that both the global (GLS, LVEF) and regional parameters (peak ${\mathrm{CS}}_{\mathrm{infarct}}$, IS) were strong independent predictors of LVR in patients with AAMI. Moreover, the peak ${\mathrm{CS}}_{\mathrm{infarct}}$ and IS might play a stronger role in predicting LVR in patients with AAMI than the global parameters do.

## Figures and Tables

**Table 1:** Demographic and clinical characteristics of the included patients with AAMI.

	All (n = 116)	Non-LVR (n = 77)	LVR (n = 39)	*p*-Value
Age, year	58.22 ± 12.30	56.96 ± 12.95	60.72 ± 10.62	0.121
Male, n (%)	100 (86.20)	67 (87.00)	33(84.60)	0.724
BMI, kg/m^2^	23.52 ± 4.24	23.07 ± 4.87	24.40 ± 2.44	0.110
Heat rate, bpm	72.75 ± 8.57	72.26 ± 8.25	73.72 ± 9.20	0.389
Pain to balloon time, h	11.12 (7.13,15.20)	10.67 (7.51,14.56)	12.02 (5.75,16.20)	0.916
CHD risk factors, n (%)				
Smoking	65 (56.00)	43 (54.80)	22 (56.40)	0.954
Hypertension	59 (50.90)	39 (50.60)	20 (51.30)	0.949
Hyperlipidemia	44 (37.9)	29 (37.70)	15 (38.50)	0.933
Diabetes	53 (45.70)	34 (44.20)	19 (48.70)	0.641
Killip classification on admission, n (%)				0.122
1	95 (81.90)	67 (87.00)	28 (71.80)	
2	16 (13.80)	8 (10.40)	8 (20.50)	
3–4	5 (4.30)	2 (2.60)	3 (7.70)	
Peak hs-cTnI, ug/L	48.15 ± 28.08	38.40 ± 26.30	67.40 ± 20.74	**<0.001**
Peak pro-BNP, ng/L	799.30 (476.00,1796.50)	724.30 (427.35,1628.00)	1066.00 (561.10,2400.50)	0.168
TIMI flow pre-PPCI, n (%)				0.943
0	71 (61.20)	46 (59.70)	25 (64.10)	
1–2	28 (24.20)	19 (24.70)	9 (23.10)	
3	17 (14.70)	12 (15.60)	5 (12.80)	
TIMI flow post-PPCI, n (%)				0.123
0–2	11 (9.50)	5 (6.50)	6 (15.40)	
3	105 (90.5)	72 (93.50)	33 (84.60)	
Medication, n (%)				
Statin	101 (87.10)	66 (85.70)	35 (89.70)	0.541
β-blocker	110 (94.80)	73 (94.80)	37 (94.90)	0.988
ACEI/ARB	54 (46.60)	35 (45.50)	19 (48.70)	0.739
Diuretic	54 (46.60)	34 (44.20)	20 (51.30)	0.467
ARNI	31 (26.70)	18 (23.40)	13 (33.30)	0.252
Nitrates	36 (31.00)	24 (31.20)	12 (30.80)	0.965

AAMI, acute anterior myocardial infarction; LVR, left ventricular remodeling; BMI, body mass index; CHD, coronary heart disease; hs-cTnI, hypersensitive serum cardiac troponin I; pro-BNP, pro-brain natriuretic peptide; TIMI, thrombolysis in myocardial infarction; PPCI, primary percutaneous coronary intervention; ACEI, angiotensin converting enzyme inhibitor; ARB, angiotensin II receptor blocker; ARNI, angiotensin receptor-neprilysin inhibitor; *p*-values of factors with bold values are less than 0.05.

**Table 2:** Intergroup comparison of CMR indexes in patients with AAMI.

	All (n = 116)	Non-LVR (n = 77)	LVR (n = 39)	*p*-Value
LVEDV, mL	155.46 ± 32.95	156.15 ± 36.17	154.12 ± 25.79	0.756
LVESV, mL	83.84 ± 21.36	81.15 ± 22.22	89.15 ± 18.69	0.056
LVEF, %	46.32 ± 6.35	48.34 ± 5.87	42.31 ± 5.32	**<0.001**
GLS, %	−10.15 ± 3.13	−11.26 ± 2.58	−7.96 ± 2.98	**<0.001**
GCS, %	−14.09 ± 3.58	−15.10 ± 3.28	−12.08 ± 3.35	**<0.001**
GRS, %	20.36 ± 5.42	21.03 ± 5.80	19.05 ± 4.38	0.063
Peak ${\mathrm{CS}}_{\mathrm{infarct}}$,%	−7.34 ± 3.88	−8.98 ± 3.42	−4.09 ± 2.43	**<0.001**
Peak ${\mathrm{CS}}_{\mathrm{remote}}$,%	−17.10 ± 3.39	−17.00 ± 3.20	−17.32 ± 3.78	0.634
Peak ${\mathrm{RS}}_{\mathrm{infarct}}$,%	11.61 ± 6.52	14.17 ± 6.14	6.57 ± 3.73	**<0.001**
Peak ${\mathrm{RS}}_{\mathrm{remote}}$,%	27.60 ± 7.01	27.78 ± 6.67	27.26 ± 7.73	0.710
IS, %LVMM	20.24 ± 8.91	17.30 ± 8.75	26.05 ± 5.91	**<0.001**

CMR, cardiovascular magnetic resonance; AAMI, acute anterior myocardial infarction; LVR, left ventricular remodeling; LVEDV, left ventricular end-diastolic volume; LVESV, left ventricular end-systolic volume; LVEF, left ventricular ejection fraction; GLS, global longitudinal strain; GCS, global circumferential strain; GRS, global radial strain; ${\mathrm{CS}}_{\mathrm{infarct}}$, circumferential strain in infarcted segments; ${\mathrm{CS}}_{\mathrm{remote}}$, circumferential strain in remote non-infarcted segments; ${\mathrm{RS}}_{\mathrm{infarct}}$, radial strain in infarcted segments; ${\mathrm{RS}}_{\mathrm{remote}}$, radial strain in remote non-infarcted segments; IS, infarct size; LVMM, left ventricular myocardial mass. Significant *p*-values (*p* < 0.05) marked in bold.

**Table 3:** Logistic regression analysis of predictors for LVR.

Parameters	Univariate Analysis		Multivariate Analysis	
	OR (95%CI)	*p*-Value	OR (95%CI)	*p*-Value
Sex, n (%)				
Male	Reference			
Female	1.218 (0.386, 3.575)	0.724		
Age, year	1.026 (0.994, 1.062)	0.122		
BMI, kg/m^2^	1.103 (0.989, 1.260)	0.116		
HR, bpm	1.020 (0.975, 1.069)	0.386		
Pain to balloon time, h	1.006 (0.949, 1.065)	0.839		
CHD risk factors, n (%)				
Hypertension	1.026 (0.474,2.226)	0.949		
Hyperlipidemia	1.034 (0.463,2.277)	0.933		
Smoker	1.023 (0.471,2.242)	0.954		
Diabetes	1.201 (0.553,2.610)	0.641		
Killip classification on admission, n (%)				
1	Reference			
2	2.393 (0.806, 7.132)	0.112		
3–4	3.569 (0.566, 28.381)	0.174		
TIMI flow post-PPCI				
0–2	2.618 (0.739,9.678)	0.133		
3	Reference			
Peak pro-BNP, ng/L	1.000 (0.999,1.000)	0.523		
Peak hs-cTnI, ug/L	1.045 (1.027,1.067)	**<0.001**		
CMR parameters				
LVEF, mL	0.839 (0.771,0.903)	**<0.001**	0.882 (0.777, 0.990)	**0.038**
GLS, %	1.634 (1.354,2.043)	**<0.001**	1.347 (1.031,1.818)	**0.039**
GCS, %	1.339 (1.168,1.572)	**<0.001**		
GRS, %	0.931 (0.861,1.003)	0.062		
Peak ${\mathrm{CS}}_{\mathrm{infarct}}$, %	1.873 (1.499,2.419)	**<0.001**	1.726 (1.365, 2.354)	**<0.001**
Peak ${\mathrm{CS}}_{\mathrm{remote}}$, %	0.972 (0.865,1.090)	0.631		
Peak ${\mathrm{RS}}_{\mathrm{infarct}}$, %	0.694 (0.582,0.794)	**<0.001**		
Peak ${\mathrm{RS}}_{\mathrm{remote}}$, %	0.989 (0.935,1.046)	0.707		
IS, %LVMM	1.156 (1.090,1.241)	**<0.001**	1.098 (1.016,1.202)	**0.027**

LVR, left ventricular remodeling; BMI, body mass index; HR, heart rate; CHD, coronary heart disease; TIMI, thrombolysis In myocardial infarction; PPCI, primary percutaneous coronary intervention; pro-BNP, pro-brain natriuretic peptide; hs-cTnI, hypersensitive serum cardiac troponin I; CMR, cardiovascular magnetic resonance; LVEF, left ventricular ejection fraction; GLS, global longitudinal strain; GCS, global circumferential strain; GRS, global radial strain; ${\mathrm{CS}}_{\mathrm{infarct}}$, circumferential strain in infarcted segments; ${\mathrm{CS}}_{\mathrm{remote}}$, circumferential strain in remote non-infarcted segments; ${\mathrm{RS}}_{\mathrm{infarct}}$, radial strain in infarcted segments; ${\mathrm{RS}}_{\mathrm{remote}}$, radial strain in remote non-infarcted segments; IS, infarct size; LVMM, left ventricular myocardial mass. Significant *p*-values (*p* < 0.05) marked in bold.

## Data Availability

The data presented in this study are available on request from the corresponding author on reasonable request.

## Supplementary Materials

The following supporting information can be downloaded at: https://www.mdpi.com/article/10.3390/diagnostics12112780/s1, Table S1: Intraclass correlation coefficient of CMR strain analysis; Figure S1: Bland–Altman analysis of LV strain indexes; Figure S2: Receiver operating characteristic analysis for predication of LVR after PPCI.
