# Supplementary material for: Predictive Value of Cardiac Magnetic Resonance for Left Ventricular Remodeling of Patients with Acute Anterior Myocardial Infarction
Source: Diagnostics (Basel). 2022 Nov 14;12(11):2780. doi: 10.3390/diagnostics12112780 (PMC9689537; doi:10.3390/diagnostics12112780)
Supplement: Supplementary file 1 [file diagnostics-12-02780-s001.zip › diagnostics-2026975-supplementary.pdf]

diagnostics MDPI

Supplementary Table S1. Intraclass correlation coefficient of CMR strain analysis.

CMR Strain Indexes	Intra-Observer	Inter-Observer
GLS, %	0.972(0.890,0.993）	0.944(0.803,0.986）
GCS, %	0.916(0.676,0.979）	0.919(0.690,0.980）
GRS, %	0.896(0.615,0.974）	0.881(0.490,0.997)
Peak $CS_{infarct}$,%	0.939(0.783,0.984）	0.908(0.678,0.976）
Peak CS_{remote},%	0.972(0.894,0.993）	0.892(0.631,0.972）
Peak $RS_{infarct}$,%	0.887(0.620,0.970）	0.911(0.515,0.980）
Peak RS_{remote},%	0.943(0.480,0.988）	0.930(0.401,0.986）

CMR, cardiovascular magnetic resonance; GLS, global longitudinal strain; GCS, global circumferential strain; GRS, global radial strain; $CS_{infarct}$, circumferential strain in infarcted segments; CS_{remote}, circumferential strain in remote non-infarcted segments; $RS_{infarct}$, radial strain in infarcted segments; RS_{remote}, radial strain in remote non-infarcted segments.

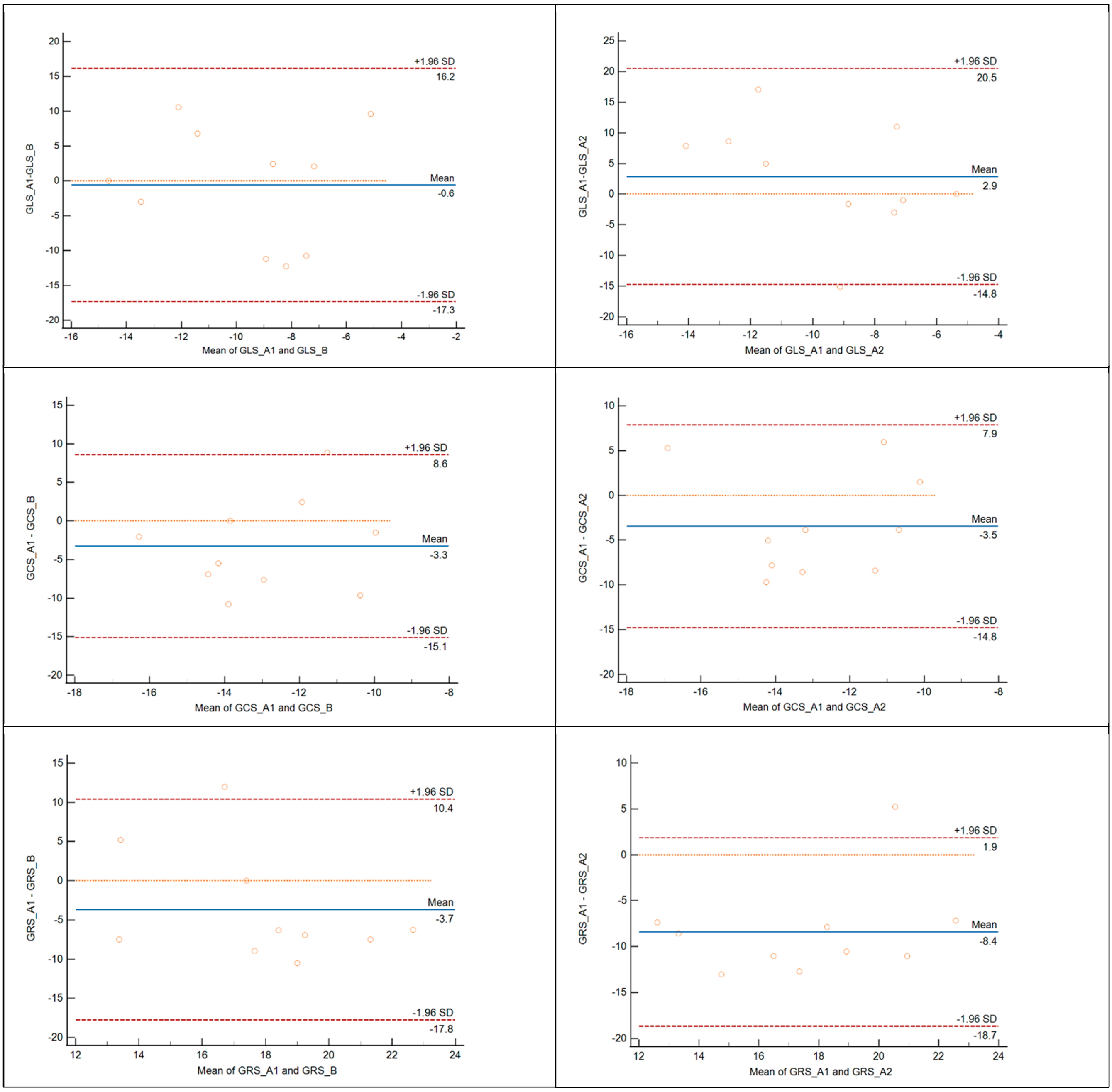

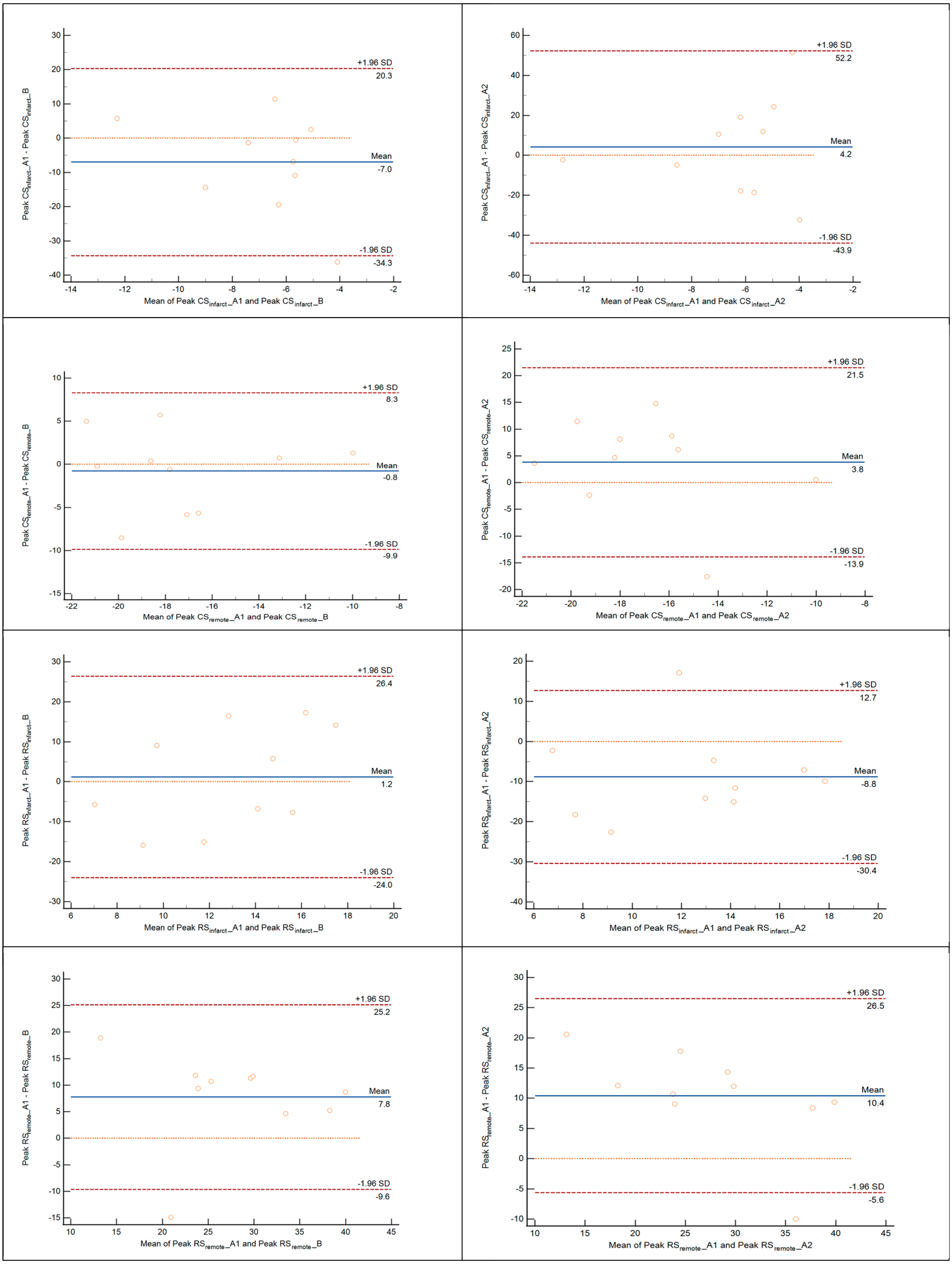

Supplementary Figure S1. Bland-Altman analysis of LV strain indexes.

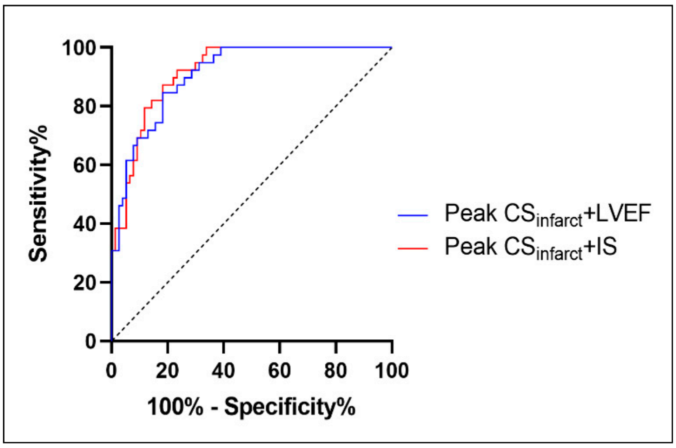

Supplementary Figure S2. Receiver operating characteristic analysis for predication of LVR after PPCI. $CS_{infarct}$ + IS had similar the area under the ROC with $CS_{infarct}$ + LVEF (0.909, 95% CI: 0.858–0.959 $p < 0.001$ *vs* 0.917, 95%CI:0.870–0.965, $p < 0.001$), LVR, left ventricular remodeling; PPCI, primary percutaneous coronary intervention; $CS_{infarct}$, circumferential strain in infarcted segments; IS, infarct size; ROC, receiver operating characteristic; LVEF, left ventricular ejection fraction.
